# A DNA Barcode Inventory of Austrian Dragonfly and Damselfly (Insecta: Odonata) Species

**DOI:** 10.3390/insects16101056

**Published:** 2025-10-16

**Authors:** Lukas Zangl, Iris Fischer, Marcia Sittenthaler, Andreas Chovanec, Patrick Gros, Werner Holzinger, Gernot Kunz, Andrea Lienhard, Oliver Macek, Christoph Mayerhofer, Marija Mladinić, Martina Topić, Sylvia Schäffer, Kristina M. Sefc, Christian Sturmbauer, Elisabeth Haring, Stephan Koblmüller

**Affiliations:** 1 Institute of Biology, University of Graz, Universitätsplatz 2, 8010 Graz, Austria; gernot.kunz@gmail.com (G.K.); lienhard.andrea@gmx.at (A.L.); marija.mladinic.m@gmail.com (M.M.); martinatopic99@gmail.com (M.T.); sylvia.schaeffer@uni-graz.at (S.S.); kristina.sefc@uni-graz.at (K.M.S.); christian.sturmbauer@uni-graz.at (C.S.); 2 Central Research Laboratories, Natural History Museum Vienna, Burgring 7, 1010 Vienna, Austria; iris.fischer@nhm.at (I.F.); marcia.sittenthaler@nhm.at (M.S.); oliver.macek@nhm.at (O.M.); elisabeth.haring@nhm.at (E.H.); 3 Department of Evolutionary Biology, University of Vienna, Djerassiplatz 1, 8010 Vienna, Austria; 4 Federal Ministry of Agriculture and Forestry, Climate and Environmental Protection, Regions and Water Management, Marxergasse 2, 1030 Vienna, Austria; andreas.chovanec@bmluk.gv.at; 5 Haus der Natur Museum, Biodiversity Center, Museumsplatz 5, 5020 Salzburg, Austria; patrick.gros@hausdernatur.at (P.G.); christoph.mayerhofer.92@gmail.com (C.M.); 6 ÖKO Team, Institut für Tierökologie und Naturraumplanung, Bergmanngasse 22, 8010 Graz, Austria; holzinger@oekoteam.at; 7 Universalmuseum Joanneum, Studienzentrum Naturkunde, Weinzöttlstraße 16, 8045 Graz, Austria; 8 Austrian Red Cross, Blood Service for Vienna, Lower Austria and Burgenland, R&D, Wiedner Hauptstraße 32, 1040 Vienna, Austria; 9 Department of Biology, Faculty of Science, University of Zagreb, Rooseveltov trg 6, 10000 Zagreb, Croatia; 10 Research Group Zoology: Biodiversity & Toxicology, Centre for Environmental Sciences, Hasselt University, Agoralaan Gebouw D, 3590 Diepenbeek, Belgium

**Keywords:** Austria, biodiversity, DNA barcoding, Odonata, *COI*, *16*S

## Abstract

**Simple Summary:**

Dragonflies and damselflies are good indicators of the health of rivers, ponds, and wetlands. In Austria, 78 species of these insects have been recorded. While we know a lot about where they live and how threatened they are, genetic data have been largely lacking. This study begins to fill that gap by creating a DNA reference library for Austrian dragonflies and damselflies. We collected many dragonfly and damselfly individuals from across Austria and sequenced two key DNA markers: *COI*, commonly used for species identification, and *16S*, which is often used to detect species from environmental samples like water (a method known as eDNA metabarcoding). More than 90% of the samples were successfully identified using both markers. Some challenges appeared with closely related species, where the genetic differences are very small, but overall, the results show that both markers work well for identifying most species. This new reference DNA barcode library will support future biodiversity monitoring and conservation work, especially through non-invasive eDNA methods that help track species without needing to see or catch them.

**Abstract:**

Dragonflies and damselflies are important indicator species for quality and health of (semi-)aquatic habitats. Hitherto, 78 species of Odonata have been reported for Austria. Ecological data, Red List assessments, and a dragonfly association index exist, but population- and species-level genetic data are largely lacking. In this study, we establish a comprehensive reference DNA barcode library for Austrian dragonflies and damselflies based on the standard barcoding marker *COI*. Because of the increasing significance of environmental DNA (eDNA) analyses, we also sequenced a segment of the mitochondrial *16S* rRNA gene, a marker often used in eDNA metabarcoding approaches. In total, we provide 786 new *COI* barcode sequences and 867 new *16S* sequences for future applications. Sequencing success was >90 percent for both markers. Identification success was similar for both markers and exceeded 90 percent. Difficulties were only encountered in the genera *Anax* Leach, 1815, *Chalcolestes* Kennedy, 1920, *Coenagrion* Kirby, 1890 and *Somatochlora* Selys, 1871, with low interspecific genetic distances and, consequently, BIN (barcode index number) sharing. In *Anax*, however, individual sequences clustered together in species-specific groups in the *COI* tree. Irrespective of these challenges, the results suggest that both markers perform well within most odonate families in terms of sequencing success and species identification and can be used for reliably delimiting Austrian species, monitoring, and eDNA approaches.

## 1. Introduction

The hemimetabolous insect order Odonata comprises three suborders, two of them—Anisoptera (dragonflies) and Zygoptera (damselflies)—are represented by 143 species throughout Europe [1,2,3]. Their relatively large body size and attractive coloration as well as their fascinating biology make them appealing to nature enthusiasts and scientists and have led to a relatively comprehensive knowledge about their diversity, distribution, reproductive behavior, and environmental requirements [1,3,4,5,6,7,8,9]. Based on this advanced state of knowledge, particularly about their amphibiotic life history and ecological preferences, Odonata are also used for environmental assessments of (semi-)aquatic habitats and monitoring within the European Water Framework Directive [10]. For Austria, several indices based on Odonata have been developed for assessing the ecological status of different aquatic habitats and the evaluation of restoration measures [11,12,13]. All this work has also led to fairly up-to-date Red List assessments and national checklists. According to the latest version of the checklist of Austrian odonates [14], 78 species belonging to nine families were recorded in Austria, of which 44 are listed in one of the three “threatened” categories in the Red List from 2006 [15]. These numbers, however, will soon be updated as the new version of the Red List for Austria is currently in preparation. Furthermore, 16 species are listed in the Annexes II and/or IV of the Habitats Directive 92/43/EEC by the European Union [2,16]. Contrasting this comprehensive knowledge about their general biology, comparatively little genetic data was available for Odonata until recently. Most of the existing genetic data was generated in the context of phylogenetic studies focusing mainly on the genus/family level [8,17,18]. In addition, character-based DNA barcoding was proposed for species discovery in odonates [19]. However, until recently, genetic reference data for national or Europe-wide species assemblages of Odonata were scarce and incomplete [20]. Those data were generated only recently following the progression of DNA barcoding becoming widely used in biodiversity research and species identification (e.g., [21,22,23,24,25,26]). The first comprehensive European odonate reference data were made publicly available in 2021 [3,27,28]. However, since geographic coverage increases species identification success as well as accuracy of genetic diversity estimation [29], more DNA sequence data of European dragonflies and damselflies is needed. This especially applies in the light of increasing (non-invasive) DNA-based approaches to study and/or monitor biological diversity, i.e., through DNA extracted from exuviae [30] or environmental DNA (eDNA) metabarcoding approaches [31,32]. These approaches may either investigate whole communities [33] or target specific species of interest [32]. However, contrary to classical DNA barcoding based on the mitochondrial cytochrome c oxidase subunit 1 gene (*COI*) as the standard marker for animal taxa, the marker of choice for (eDNA) metabarcoding is still open for discussion and may be dependent on the research question [34,35]. While *COI* has become the standard marker for metabarcoding of insects (e.g., [36,37,38], (eDNA) metabarcoding studies that target a larger taxonomic diversity including both invertebrates and vertebrates often use other markers that contain less primer site variation even across higher taxonomic levels, thus increasing the likelihood of successful amplification of all components of bulk samples containing large taxonomic diversity [34,39]. One occasionally used marker for assessing aquatic biodiversity, often in combination with *COI*, is a region of the mitochondrial *16S* rRNA gene [40,41,42,43,44].

In this study, we present a comprehensive and almost complete reference *COI* DNA barcode inventory of Austrian Odonata as well as their respective *16S* sequences. We compare the performance of both markers with regard to sequencing success, species identification, and discrimination and ponder the suitability for eDNA approaches.

## 2. Materials and Methods

The present study includes sequences of 892 specimens (Table S1) from all nine federal states of Austria covering all extant native odonate families. From 2017 to 2020, 839 of them were collected under the following permits: ABT13-53S-7/1996-156, ABT13-53W-50/2018-2, ABT13-198250/2020-9, RU5-BE-1489/001-2018, RU5-BE-64/018-2018, RU5-BE-1489/002-2021, MA22-169437/2017, A4/NN.AB-10097-5-2017, A4/NN.AB-10200-5-2019, SP3-NS-3375/2019 (005/2019), FE3-NS-2650/2019 (009/2019), N-2020-68581/4-Has, and 08-NATP-845/1-2019 (007/2019). Imagines were caught with an insect net and larvae were collected by hand. Six specimens (*Aeshna viridis* Eversmann, 1836 and *Stylurus flavipes* (Charp, 1825), three specimens each, collected between 1968 and 1988) were obtained from the entomological collection of the Natural History Museum Vienna and an additional 53 sequences of Austrian odonates (24 sequences from [45], and 29 sequences from [3]) were retrieved from BOLD. Specimens were photographed and tissue samples (legs) were stored in pure ethanol. The specimens, either mounted dry or preserved in a water–ethanol–glycerol solution (5:85:10), were transferred to scientific collections. All collection and storage metadata of the 892 specimens analyzed here are available on BOLD (https://dx.doi.org/10.5883/DS-BAAZ).

Tissue samples for genetic investigations were processed at the Central Research Laboratories of the Natural History Museum Vienna (NHM Vienna) and the University of Graz. Sequencing efforts followed a three-pronged approach: (1) For the *COI* gene, we tried to amplify major parts or even the complete *COI* gene in order to potentially gain additional species-specific genetic information not contained in the Folmer region constituting the ‘classical’ DNA barcode sequence. (2) Alternatively, at least the Folmer region of the *COI* gene was recovered, and (3) a partial fragment of the mitochondrial 16S (*16S*) gene was sequenced for all newly caught specimens as another widely used eDNA marker. As protocols varied between the two laboratories, procedures for generating sequences are described separately in the following paragraphs.

For samples processed at the NHM Vienna: For DNA extraction, the coxa and a small piece of the femur were used as starting material. For freshly collected samples, a standard DNA extraction was performed with the DNeasy Blood and Tissue Kit (Qiagen, Hilden, Germany) according to the manufacturer’s protocol, with a final elution volume of 60 µL AE buffer. For DNA extraction of museum specimens (dry collection), as lower DNA yields were expected, the QIAamp DNA Micro Kit (Qiagen, Hilden, Germany) was used according to the manufacturer’s protocol with a final elution volume of 40 µL AE buffer. For *COI*, mostly PCR primers binding close to or within the flanking tRNA genes (tRNA Tyr gene and tRNA Leu gene) were used, amplifying a ~1600 bp long fragment at once (Table 1). Alternatively, a *COI* sequence was generated by combining two shorter, overlapping amplicons (between ~500 and 800 bp), which were generated in separate PCRs with different primers depending on the species. All primers used for PCR are listed in Table 1. Regardless of whether the final *COI* sequence was amplified in one piece or composed of two amplicons, the consensus sequences included the ~650 bp long standard DNA barcoding region (the Folmer region). PCRs of the 1600 bp long fragment were performed with the Qiagen Taq Polymerase (Qiagen, Hilden, Germany) in a volume of 50 μL containing 0.5 μL Taq polymerase (5 units/μL), 5 μL of 10 × PCR Buffer (Qiagen, Hilden, Germany), 10 μL of 5 × Q-Solution (Qiagen, Hilden, Germany), 1.5 mM MgCl_2_, 2.5 mM dNTP Mix, 0.5 μM of each primer, and 1 μL DNA template. The PCR cycling protocol included an initial denaturation at 94 °C for 3 min, followed by 35 cycles of denaturation at 94 °C for 60 s, annealing (for primer-specific annealing temperatures see Table 1) for 30 s and extension at 72 °C for 60 s. The final step was an extension at 72 °C for 10 min. For PCR amplification of the partial *16S* fragment, the primer pair 16S-Odo-F1/16S-Odo-R2 (Table 1) resulting in an amplicon length of ~560 bp (60 °C annealing temperature) was used. PCRs with primers for *16S* and the shorter overlapping *COI* amplicons were performed in a volume of 25 µL containing 0.25 µL Qiagen Taq Polymerase (5 units/µL; Qiagen, Hilden, Germany), 2.5 µL 10 × PCR Buffer (Qiagen, Hilden, Germany), 5 µL 5 × Q-Solution (Qiagen, Hilden, Germany), 1.5 µL of MgCl_2_ (1.5 mM), 0.5 µL of dNTP Mix (2.5 mM), 0.5 µL of each primer (50 pmol/µL), and 1 µL template DNA. The PCR cycling protocol was the same as for the *COI* amplification (see above). For museum specimens (*Aeshna viridis,* and *Stylurus flavipes*), however, only the 560 bp long *16S* fragment and for *COI* the standard barcoding region, using PCR primer combinations amplifying two shorter (~200 and ~600 bp), overlapping fragments (ODO_LCO1490d/CO1-OdoCol-R1 and CO1-Zyg-F1/ODO_HCO2198d [45,46], both with an annealing temperature of 57 °C) were amplified. PCR reactions with DNA stemming from museum material were performed with the Multiplex PCR Kit (Qiagen, Hilden, Germany) in a volume of 25 µL, containing 12.5 µL Multiplex PCR Master Mix, 0.5 µM of each primer, and 2 µL of template DNA. The PCR cycling protocol included an initial denaturation at 95 °C for 15 min, followed by 40 cycles of denaturation at 94 °C for 30 s, annealing for 90 s and extension at 72 °C for 60 s. The final step was an extension at 72 °C for 10 min. For all PCR reactions, PCR success was checked on 1% agarose gels, and PCR products were subsequently purified with the QIAquick PCR Purification Kit (Qiagen, Hilden, Germany). All samples were sequenced in both directions (Microsynth, Balgach, Switzerland) using the PCR primers as well as two additional internal primers (Table 1). For the smaller fragments (*16S* and *COI*, from museum specimens), only PCR primers were used for sequencing.

For samples processed at the University of Graz: Extraction of whole genomic DNA followed a rapid Chelex protocol described in [47]. Subsequent PCRs for the amplification of *COI* were conducted in a total of 10 µL containing 7.05 µL of water, 1 µL of 10 × buffer BioTherm containing 15 mM MgCl_2_ (Gene Craft, Lüdinghausen, Germany), 0.35 µL of 1 mM dNTPs (1 mM), 0.1 µM of each primer, 0.5 units of BioTherm DNA polymerase (Gene Craft, Lüdinghausen, Germany), and 1 µL of template DNA using the primers ODO_LCO1490d, ODO_HCO2198d, Tyr-Odo-F, and Leu-Odo-R [45,46] (Table 1). Cycling conditions were as follows: three minutes of initial denaturation at 95 °C, followed by 45 cycles of denaturation at 95 °C for 30 s, varying annealing temperatures (Table 1) for 30 s and extension at 72 °C for one minute as well as a final extension phase at 72 °C for seven minutes. Identical settings were also used for amplifying the *16S* fragment with the only exception being the primers which were the same as being used by the NHM Vienna. Success of PCRs were checked via a 2% agarose gel electrophoresis and successful PCR products were cleaned using Exo-Sap-IT Express PCR Product Cleanup (Applied Biosystems by Thermo Fisher Scientific, Waltham, MA, USA). Bidirectional Sanger sequencing was performed following [48] using the primers ODO_LCO1490d and ODO_HCO2198d or Tyr-Odo-F and Leu-Odo-R for *COI* and 16S-Odo-F1 and 16S-Odo-R2 for *16S* (Table 1).

Trace files were checked with MEGA version 6 [49] and forward and reverse reads were combined to consensus sequences and aligned using the built-in MUSCLE algorithm. All newly generated *COI* and *16S* sequences were uploaded to BOLD (https://dx.doi.org/10.5883/DS-BAAZ) and subjected to subsequent clustering (*COI* and *16S*), genetic distance, and BIN assignment analyses (only *COI*). Furthermore, additional sequences of dragonfly and damselfly species from Austria available on BOLD [3,30,45] were appended to the dataset resulting in alignments of 839 *COI* sequences (658/1536 bp) and 867 partial *16S* sequences (547 bp), respectively (Table S1).

The ‘Taxon ID Tree’ tool implemented on BOLD (applying the Kimura 2 Parameter distance model and the BOLD aligner including all codon positions and the pairwise deletion option) was used to illustrate species-specific clustering on separate neighbor joining trees (NJ) for Anisoptera and Zygoptera based on sequence similarity of partial *COI* sequences (covering the Folmer region). Maximum intra- and minimum interspecific genetic distances were calculated using the ‘Distance Summary’ and ‘Barcode Gap Analysis’ tools (K2P distance model, complete deletion of ambiguous characters or missing data, and BOLD aligner), also implemented on BOLD. Furthermore, assignment of individual *COI* sequences to BINs was also checked on BOLD. Additionally, separate alignments for Anisoptera and Zygoptera containing only full-length *COI* sequences (>1000 bp) were created in MEGA and used for NJ tree inference and for comparison with the shorter dataset covering the Folmer region (using the pairwise deletion option for ambiguous or missing data). These datasets, however, did not contain all available species anymore as full-length sequences were not available for all species (Anisoptera: 194 sequences, 38 species; Zygoptera: 113 sequences, 21 species). For *16S*, sequences of Anisoptera and Zygoptera were aligned separately with MEGA and subsequently used for NJ tree inference and for mean between-group (species) genetic distance calculation. Finally, the overall performance of the two markers (as well as the standard and full-length dataset for *COI*) was compared for the distinct species based on sequencing success (ratio samples/sequences) and discriminatory power (resolution in phylogram).

## 3. Results

**Figure 1:**
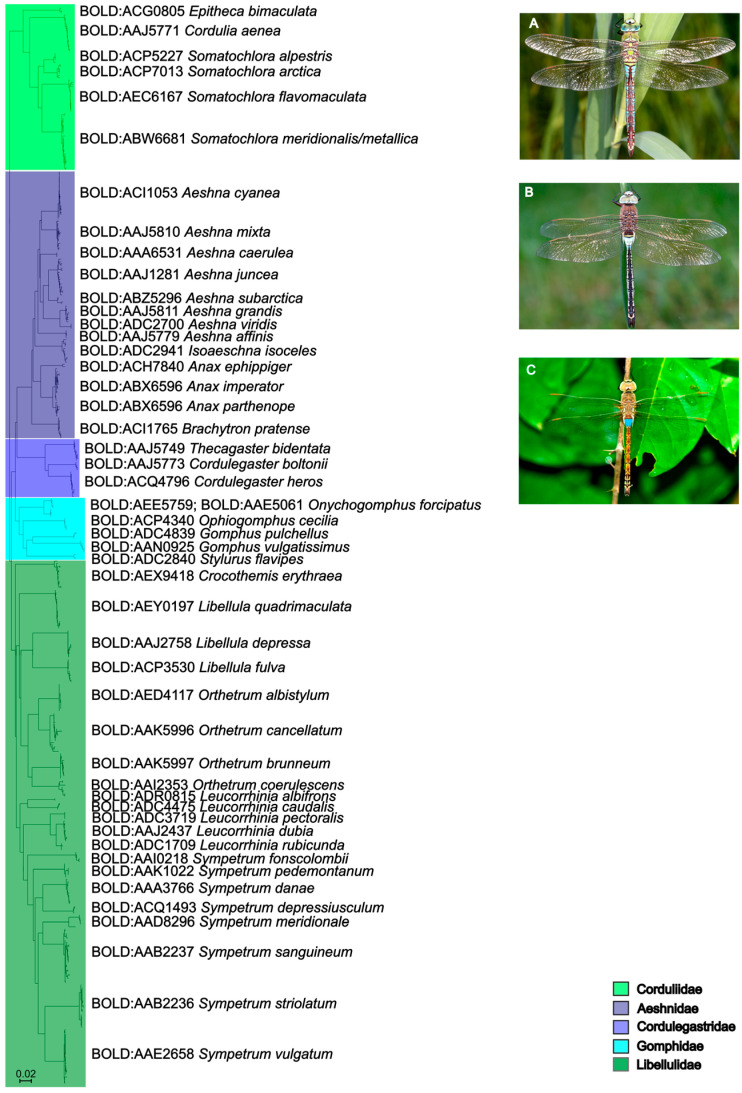
NJ tree based on 481 *COI* sequences of Austrian dragonflies (Odonata: Anisoptera). © Pictures of *Anax imperator* (**A**) and *A. parthenope* (**B**) provided by Gernot Kunz; picture of *A. ephippiger* (**C**) from Avtor: Sandesh Gawas—this image was uploaded from observation number 346,695 at India Biodiversity Portal, a source of nature observations in India. License: CC BY 3.0; URL: https://commons.wikimedia.org/w/index.php?curid=56124157 (accessed on 15 May 2025).

**Figure 2:**
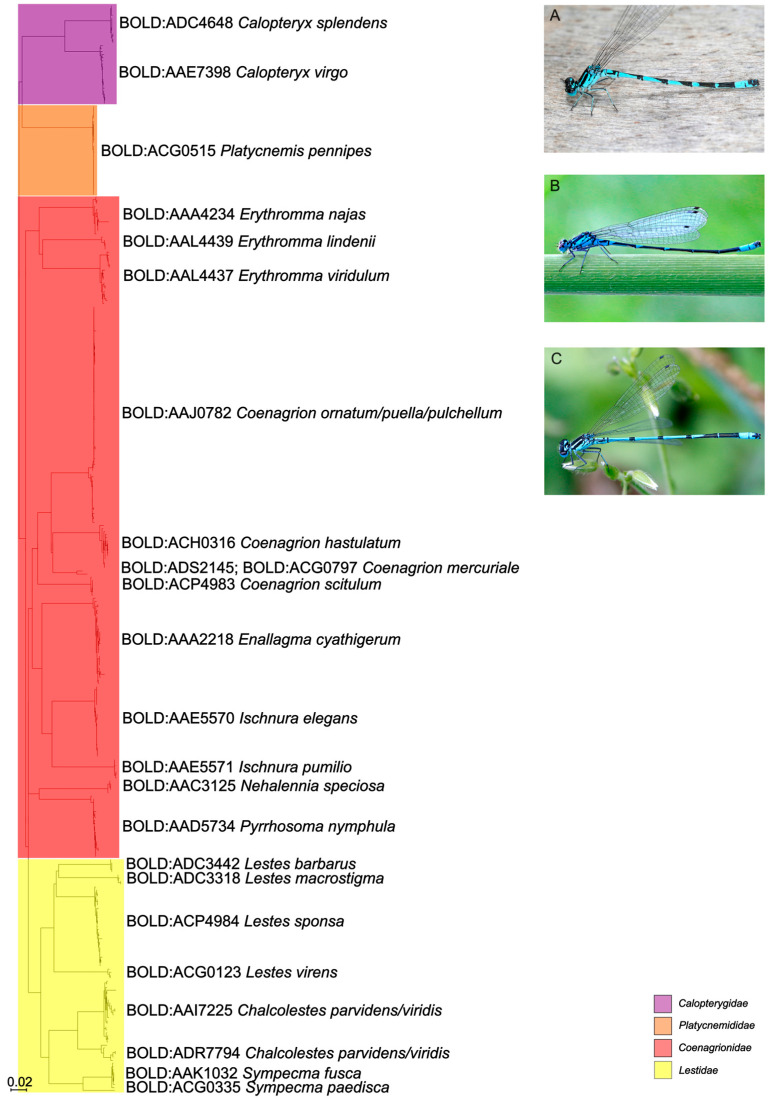
NJ tree based on 358 *COI* sequences of Austrian damselflies (Odonata: Zygoptera). © Pictures of *Coenagrion ornatum* (**A**), *C. puella* (**B**), and *C. pulchellum* (**C**) provided by Gernot Kunz.

In this study, 786 new *COI* DNA barcode sequences and 867 partial *16S* sequences were generated, covering all nine families, 27 genera, and 74 out of 78 species of dragonflies and damselflies reported from and/or present in Austria. All species, except *Anax ephippiger* (Burmeister, 1839) (2), *Coenagrion mercuriale* (Charpentier, 1840) (2), *Sympecma paedisca* (Brauer, 1877) (1), and *Leucorrhinia albifrons* (Burmeister, 1839) (2) were represented by at least three specimens. Only *Coenagrion hylas* (Trybom, 1889), *C. lunulatum* (Charpentier, 1840), *Lestes dryas* (Kirby, 1890), and *Sympetrum flaveolum* (Linnaeus, 1758) could not be collected or sequenced at all. Overall, the sequencing success (ratio of successfully recovered sequences compared to the overall number of specimens sampled) amounted to 94 percent of specimens for *COI* and 96 percent for *16S*. The 74 morphologically identified species were represented by 73 distinct BINs (Table 2) and 71 distinct clusters or singletons in the *COI* NJ trees based on the short fragments (Figure 1 and Figure 2). BIN sharing was detected in *Coenagrion ornatum* (Selys, 1850)/*C. puella* (Linnaeus, 1758)/*C. pulchellum* (Vander Linden, 1825)*, Anax imperator* Leach, 1815/*A. parthenope* Selys, 1939, *Somatochlora meridionalis* Nielsen, 1935/*S. metallica* (Vander Linden, 1825), and *Chalcolestes parvidens* (Vander Linden, 1825)/*C. viridis* (Vander Linden, 1825) (Figure 1 and Figure 2, Table 2). *Chalcolestes parvidens* and *C. viridis* shared two BINs (BOLD:AAI7225; BOLD:ADR7794). *Coenagrion mercuriale*, on the other hand, was represented by two different BINs (BOLD:ADS2145, BOLD:ACG0797). Furthermore, whereas *C. ornatum*/*C. puella*/*C. pulchellum, S. meridionalis*/*S. metallica* and *C. parvidens*/*C. viridis* constituted mixed clades in the NJ trees, Austrian *Anax imperator* and *A. parthenope* occupied two distinct monophyletic clades in the NJ tree and were clearly resolved as distinct entities despite their shared BIN. K2P distances within species ranged from 0 to 3.17% (10.21% in cases of deep intraspecific divergences, usually together with BIN sharing; mean: 0.55%) and between species from 2.30 to 16.91% (0% in cases of BIN sharing) resulting in a pronounced DNA barcoding gap for all species except (i) *Coenagrion ornatum/C. puella*/*C. pulchellum*, (ii) *Somatochlora meridionalis* and *S. metallica*, and (iii) *Chalcolestes parvidens* and *C. viridis* (Figure S1).

**Figure 3:**
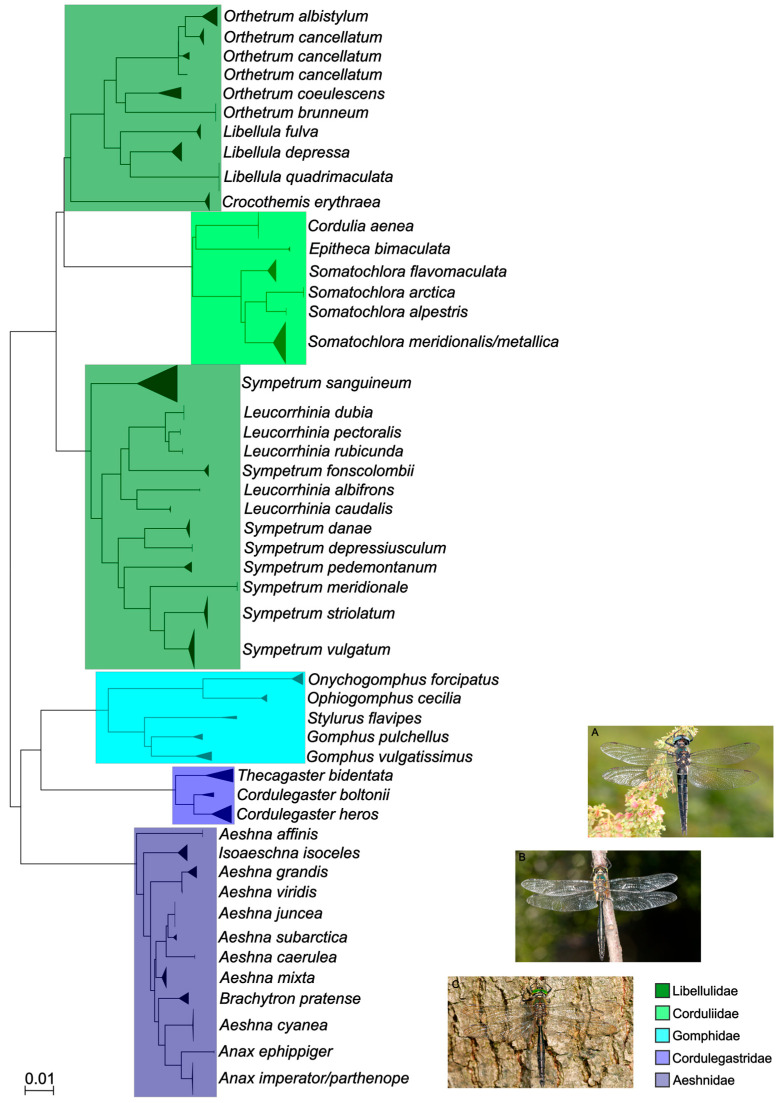
NJ tree based on 482 partial *16S* sequences of Austrian dragonflies (Odonata: Anisoptera). © Pictures of *Somatochlora alpestris* (**A**), *S. meridionalis* (**B**), and *S. metallica* (**C**) provided by Gernot Kunz.

**Figure 4:**
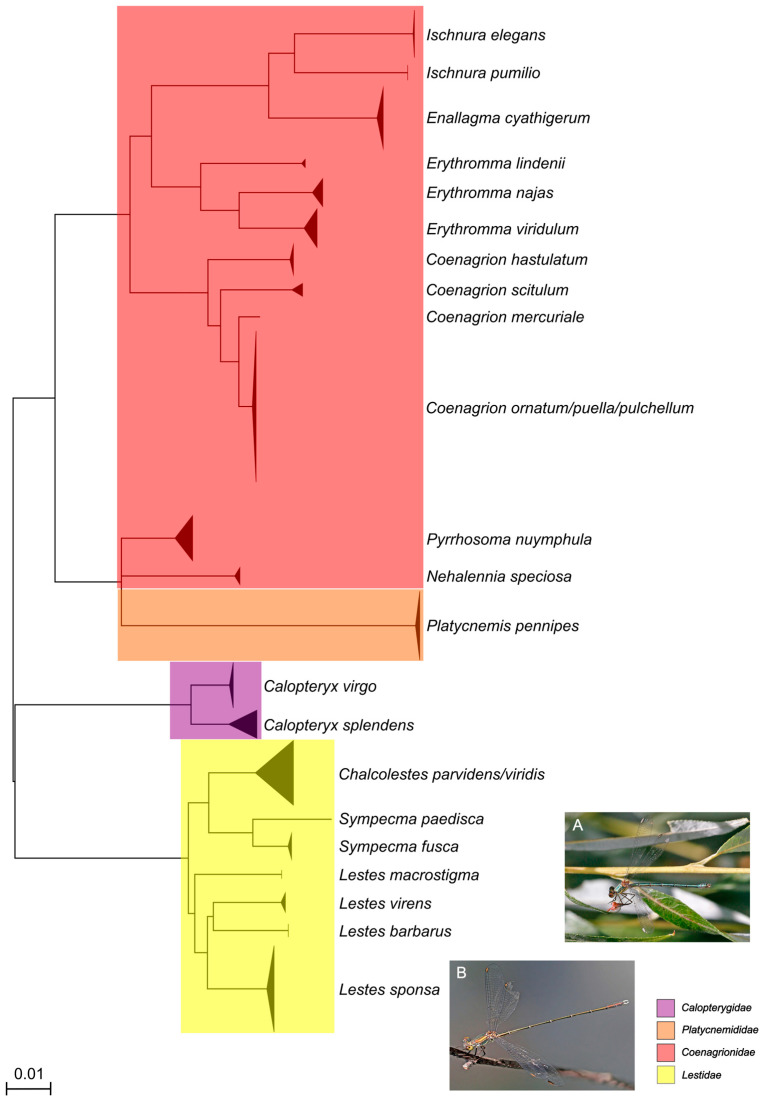
NJ tree based on 381 partial *16S* sequences of Austrian damselflies (Odonata: Zygoptera). © Pictures of *Chalcolestes viridis* (**A**) and *C. parvidens* (**B**) provided by Gernot Kunz and Charles J. Sharp—own work, from Sharp Photography, sharpphotography.co.uk, CC BY-SA 4.0, https://commons.wikimedia.org/w/index.php?curid=109369150 (accessed on 15 May 2025), respectively.

Similar results were also obtained for *16S*. *Somatochlora meridionalis/S. metallica, Anax imperator/A. parthenope, Coenagrion ornatum/C. puella/C. pulchellum*, and *Chalcolestes parvidens/C. viridis* and could not be distinguished based on their respective *16S* sequences either and were consequently forming mixed clades in the *16S* trees (Figure 3 and Figure 4). Additionally, *Orthetrum cancellatum* (Linnaeus, 1758) was not recovered as a monophyletic group, but was divided in three clades which, however, exclusively contained *O. cancellatum* sequences (Figure 3). All other species could be clearly recognized by their respective *16S* sequence and, consequently, were occupying distinct clades in the *16S* NJ tree. Mean within- and between-group (species) genetic distances (Table S2) mirrored the results of *COI* genetic distances (Table 2) although *16S* distances were approximately five times lower than *COI* distances.

The comparison between the standard (Folmer region; Figure 1 and Figure 2) and full-length *COI* dataset (Figures S2 and S3) revealed an equal performance in terms of species distinction in the NJ tree. In particular, unresolved morphospecies like in the genera of *Somatochlora*, *Chalcolestes*, or *Coenagrion* could also not be resolved with the full-length data set.

**Figure 5:**
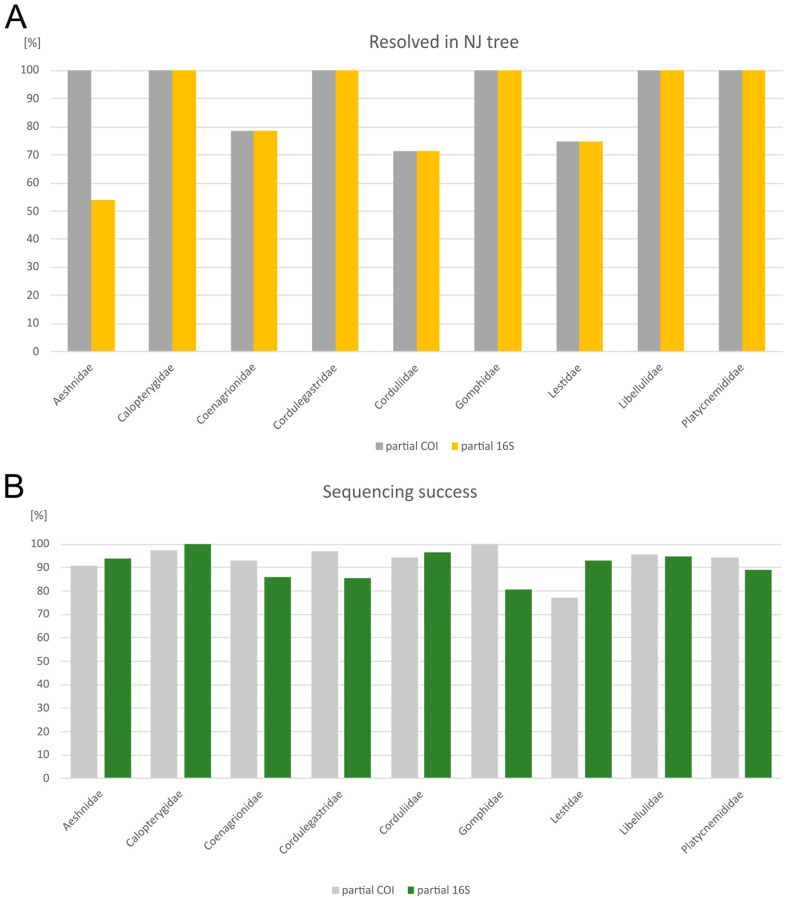
Family-level comparison of species resolved in the NJ tree (**A**) and the sequencing success (**B**) based on partial *COI* and partial *16S* sequences.

The comparison between the *COI* and the *16S* datasets revealed that, in terms of sequencing success, both markers performed almost equally well (Figure 5). The sequencing success was very high in general (mostly above 90%) with the only drop below 80% for *COI* in the family Lestidae. For species discrimination, both genetic markers performed similarly. Sequences from Calopterygidae, Cordulegastridae, Gomphidae, Libellulidae, and Platycnemididae were unambiguously assigned to morphospecies level using both *COI* and *16S*. However, *Anax imperator* and *A. parthenope* were resolved as two distinct clades sharing a single BIN in the *COI* tree, but formed a single mixed clade in the *16S* tree. Both markers failed to differentiate among *Coenagrion ornatum/puella/pulchellum, Somatochlora meridionalis/metallica*, and *Chalcolestes parvidens/viridis*.

## 4. Discussion

In this study, we compiled an almost complete genetic reference database of Austrian dragonflies and damselflies consisting of 839 *COI* barcode sequences and 867 partial *16S* sequences (Table S1). BIN assignment, genetic distance analysis, and clustering of *COI* sequences are generally in line with results obtained by [3,27,28], but a further increase in regional representation is important for reliable genetic species identification [3,20,29]. No new BINs were detected. Conflicting cases between morphological and genetic identification, which point to incidental hybridization or introgression, were generally corroborating those found by [3,27]. *Somatochlora meridionalis* and *S. metallica* shared BINs/haplotypes. Interestingly, there are already indications for potential hybridization between different (not Central European) *Somatochlora* species in the literature [50,51,52,53]. The patterns of BIN and haplotype sharing between *Somatochlora meridionalis* and *S. metallica*, however, does not necessarily imply hybridization, but could equally likely be due to incomplete lineage sorting of two recently diverged species. Indeed, the two species are morphologically very similar but also distinct [54] and closely related based on genomic data [55], and the species status of *S. meridionalis* is still disputed by some researchers [56]. Clear evidence for introgressive hybridization was found in *Chalcolestes parvidens* and *C. viridis* [3,8,27]. Unfortunately, these ambiguities could not be further resolved by adding more information from the *COI* region as we sequenced large parts or even the entire *COI* gene. Also, in *Coenagrion ornatum/puella/pulchellum* distinction at the species level was not achieved. For this group, although mtDNA data show BIN sharing across large parts of the species’ distribution range [3,27,57], nuclear data successfully separate the three species into distinct entities [27,58], corroborating the assumption that the observed BIN sharing might be due to introgressive hybridization and potentially mitochondrial capture over large parts of the species distribution ranges, rather than incomplete lineage sorting, which, in theory, could also be responsible for BIN sharing. Reports of heterospecific pairing and hybridization between *C. puella* and *C. pulchellum* have been published previously [59,60], adding to the plausibility of such a scenario. Regarding *Chalcolestes*, southeastern Austria represents the northernmost edge of the distribution range of *C. parvidens* [61], and hybrids are known from places where *C. parvidens* co-occurs with *C. viridis*, including the very eastern part of Austria [62]. Thus, hybridization followed by back-crossing might indeed be responsible for BIN-sharing between these two species.

Interestingly, while some taxa cannot be clearly resolved at species levels based on their DNA barcodes across their entire geographic ranges, our Austrian data show that accurate identification is still possible locally. For instance, on a Holarctic scale, both *Aeshna juncea* (Linnaeus, 1758) and *A. subarctica* Walker, 1908 comprise several BINs with some BIN sharing between the two species. In addition, the two species are not reciprocally monophyletic based on DNA barcodes. These patterns not only indicate geographic structure but also introgressive hybridization and or (ancient) incomplete lineage sorting, if we assume morphological identification of the specimens was correct [63]. In Austria, as for the rest of Europe [3,27], the two species comprise one single BIN each with no indication of interspecific hybridization. BIN sharing between *Anax imperator* and *A parthenope* has been shown before, with all *A. imperator* and *A. parthenope* constituting a single BIN [3,27,28] and evidence for haplotype sharing [59]. Even though, in our Austrian data, the two species also form a single BIN, they fall into two reciprocally monophyletic groups. However, considering the range-wide pattern of some haplotype sharing and phenotypic evidence for hybridization between the two species [64], DNA barcode-based species assignment in this species pair cannot be performed with 100% confidence in Austria, too.

In general, however, sequencing and species identification success based on *COI* barcode sequences was very high for most specimens confirming its applicability for genetic biodiversity assessments [3,27,65]. On the other hand, sequencing and identification success was also comparably high for *16S*, which is a marker often considered for eDNA and metabarcoding approaches [66,67,68]. Naturally, the choice of marker is dependent on the research question and aspects like the universality of the primers and the species-level resolution as well as the coverage of respective reference databases (like BOLD or NCBI GenBank) which may influence the decision [29,66,69]. Despite ongoing discussions about primer choice in eDNA studies [68,70,71], the results of this study seem to underscore the suitability of *16S* as a marker for eDNA studies in odonates, due to both the discriminatory power on the species level as well as the availability of highly conserved primer regions (Table 1).

Both, DNA barcoding as well as eDNA metabarcoding, gain increasing importance for the identification and monitoring of (rare and/or threatened) odonate species, especially with regard to non-invasive sampling [30,32,72]. Considering that Austria is home to 11 of the 16 species protected by the EU Habitats Directive (Directive 92/43/EEC, annexes II and/or IV) and that 56% of the 78 species reported from Austria fall into one of the three ‘threatened’ categories [15], proper monitoring is not only a legal obligation but a conservation necessity. For the present study, four species (*Coenagrion hylas, Coenagrion lunulatum, Lestes dryas*, and *Sympetrum flaveolum*) which have been reported from Austria until the beginning of the 21st century [72,73,74,75,76] were not found and/or could not be sampled at all and for another four species, fewer than three samples could be acquired each. To some extent, this might be indicative of various threats like changes in land use, loss of habitat, increased nutrient deposition, or more rapid desiccation (especially of bogs) due to climatic change, entailing higher temperatures and more frequent and severe droughts impacting the larvae of these species. Two of these species, *C. hylas* and *C. lunulatum*, are known to be very rare in Austria. *Coenagrion hylas* is a glacial relict species with, apart from some location in northwestern Russia, vital populations in Europe known only from a very limited area in the northern Calcareous Alps of Tyrol, Austria, and might hence be considered the rarest European zygopteran species [56,77]. *Coenagrion lunulatum* is a Euro-Siberian species, with very few Austrian records over the last decades [56,78]. The other two species, *L. dryas* and *S. flaveolum*, have an allegedly wide distribution, also in Austria [56]. Both species, however, rely on ephemeral habitats, such as flooded meadows and swampy depressions, types of habitat that have become increasingly rare in Austria. These threats may also change over time [79] and can lead to re-arrangements of regional diversity, i.e., species composition [80,81]. Some species have already been reported to expand their altitudinal and latitudinal range [80,82,83,84] as a consequence of changing environments and climate change, but since this is not possible for every species, for example, cold-adapted higher-altitude species, they could be threatened by extinction. However, predictions and evaluations of these dynamics are often difficult to make as population trends of endangered and protected species are still unclear in many parts of Europe. Furthermore, several endangered species are not even listed in the annexes of the Habitats Directive of the European Union [85]. Therefore, increased monitoring efforts are needed to track changing population trends and regional Odonata community assemblages and provide data for informed directions of conservation priorities. Approaches like the genetic identification of larvae, the non-invasive DNA barcoding of exuviae, species delimitation by taxonomic lay people, or large-scale monitoring via eDNA [30,31,32] all rely on reliable and comprehensive reference data which we have provided in this study to make them available for both scientists and conservationists alike.

## Figures and Tables

**Table 1:** List of all primers used for PCR amplification and sequencing (sequencing primer = seq. primer; internal sequencing primer = int. seq. primer.) of *COI* and partial *16S* fragments. Annealing temperature used in different primer combinations is given by T_ann_ [°C].

Primer Name	Primer Sequence 5′-3′	Gene	T_ann_ [°C]	Primer Type	Reference
Tyr-Odo-F	CTCCTATATAGATTTACAGTCT	*COI*	46–54	PCR/seq.	[45]
Leu-Odo-R	CTTAAATCCATTGCACTTTTCTGCC	*COI*	53–56	PCR/seq.	[45]
CO1-Odo-F5	TGCGACRATGRCTGTTTTC	*COI*	47	PCR/seq.	[45]
CO1-Odo-R6	TGCACTTTTCTGCCACATTAAA	*COI*	47–54	PCR/seq.	[45]
ODO_LCO1490d	TTTCTACWAACCAYAAAGATATTGG	*COI*	48–57	PCR/seq.	[46]
ODO_HCO2198d	TAAACTTCWGGRTGTCCAAARAATCA	*COI*	48–57	PCR/seq.	[46]
CO1-Zyg-F1	TTGGAGATGAYCAAATTTATAAYGT	*COI*	57	PCR/seq.	[45]
CO1-Odo-F3	GATTCTTTGGACAYCCHGAAG	*COI*	53	PCR/seq.	[45]
CO1-Odo-R1	TAATATGTGAAATTATWCCAA	*COI*	46	PCR/seq.	[45]
CO1-OdoCol-R1	CCTCCAATTATRATAGGTATWACTA	*COI*	57	PCR/seq.	Present study
CO1-Odo-R8	GTARTTTTTGATATCATTCRAT	*COI*	60	int. seq. primer	[45]
CO1-Lib-F1	TTAACAGAYCGAAATATTAATAC	*COI*	60	int. seq. primer	[45]
CO1-Odo-F1	GGWATAATTTCACATATTATTGC	*COI*	60	int. seq. primer	[45]
CO1-Odo-F4	TATGCAATARTAGCHATTGG	*COI*	60	int. seq. primer	Present study
CO1-Sym-F1	TTAACTGAYCGAAATATTAATACATC	*COI*	60	int. seq. primer	[45]
CO1-Lib-R1	CCTARAATACCAATTGCTACTAT	*COI*	60	int. seq. primer	[45]
CO1-Odo-R3	GTTTCCTTTTTACCTCTTTCTTG	*COI*	60	int. seq. primer	[45]
CO1-Odo-R4	CCAATTGCTAYTATTGCRTA	*COI*	60	int. seq. primer	Present study
16S-Odo-F1	GGTCTGAACTCAGATCATGTAAG	*16S*	50–60	PCR/seq.	Present study
16S-Odo-R2	CGCCTGTTTATCAAAAACATGTC	*16S*	50–60	PCR/seq.	Present study

**Table 2:** Information on barcode index number (BIN) assignments, number of samples per species (N), as well as maximum intra- (I_max_) and minimum interspecific K2P distance (distance to nearest neighbor, DNN) based on *COI* data, and the most closely related species (nearest neighbor) in our dataset.

Species	BIN	N	I_max_	DNN	Nearest Neighbor
Aeshnidae					
*Aeshna affinis* Vander Linden, 1820	BOLD:AAJ5779	4	0.28	7.11	*A. mixta*
*Aeshna caerulea* Strøm, 1783	BOLD:AAA6531	3	0.00	5.99	*A. cyanea*
*Aeshna cyanea* (Müller, 1764)	BOLD:ACI1053	22	0.74	5.71	*A. mixta*
*Aeshna grandis* (Linnaeus, 1758)	BOLD:AAJ5811	8	0.75	3.15	*A. viridis*
*Aeshna juncea* (Linnaeus, 1758)	BOLD:AAJ1281	20	1.55	3.50	*A. subarctica*
*Aeshna mixta* Latreille, 1805	BOLD:AAJ5810	14	0.94	5.71	*A. cyanea*
*Aeshna subarctica* Walker, 1908	BOLD:ABZ5296	4	1.03	3.50	*A. juncea*
*Aeshna viridis* Eversmann, 1836	BOLD:ADC2700	3	0.00	3.15	*A. grandis*
*Anax ephippiger* (Burmeister, 1839)	BOLD:ACH7840	2	0.85	6.63	*A. imperator*
*Anax imperator* Leach, 1815	BOLD:ABX6596	15	0.89	0.98	*A. parthenope*
*Anax parthenope* Selys, 1839	BOLD:ABX6596	7	0.84	0.98	*A. imperator*
*Brachytron pratense* (Müller, 1764)	BOLD:ACI1765	10	0.57	8.65	*A. mixta*
*Isoaeschna isoceles* (Müller, 1767)	BOLD:ADC2941	11	1.67	7.88	*A. caerulea*
Calopterygidae					
*Calopteryx splendens* (Harris, 1782)	BOLD:ADC4648	13	0.59	9.96	*C. virgo*
*Calopteryx virgo* (Linnaeus, 1758)	BOLD:AAE7398	20	0.71	9.96	*C. splendens*
Coenagrionidae					
*Coenagrion hastulatum* (Charpentier, 1825)	BOLD:ACH0316	15	1.39	10.15	*C. mercuriale*
*Coenagrion mercuriale* (Charpentier, 1840)	BOLD:ADS2145 BOLD:ACG0797	2	2.10	7.91	*C. pulchellum*
*Coenagrion ornatum* (Selys, 1850)	BOLD:AAJ0782	6	0.57	0.00	*C. puella*
*Coenagrion puella* (Linnaeus, 1758)	BOLD:AAJ0782	42	0.71	0.00	*C. pulchellum*
*Coenagrion pulchellum* (Vander Linden, 1825)	BOLD:AAJ0782	26	1.86	0.00	*C. puella*
*Coenagrion scitulum* (Rambur, 1842)	BOLD:ACP4983	10	0.58	12.22	*C. mercuriale*
*Enallagma cyathigerum* (Charpentier, 1840)	BOLD:AAA2218	30	1.33	13.50	*I. elegans*
*Erythromma lindenii* (Selys, 1840)	BOLD:AAL4439	10	0.56	15.28	*E. viridulum*
*Erythromma najas* (Hansemann, 1823)	BOLD:AAA4234	13	2.22	14.39	*E. viridulum*
*Erythromma viridulum* (Charpentier, 1840)	BOLD:AAL4437	18	2.24	14.39	*E. najas*
*Ischnura elegans* (Vander Linden, 1820)	BOLD:AAE5570	24	0.70	13.31	*I. pumilio*
*Ischnura pumilio* (Charpentier, 1825)	BOLD:AAE5571	7	0.44	13.31	*I. elegans*
*Nehalennia speciosa* (Charpentier, 1840)	BOLD:AAC3125	5	0.59	15.27	*P. nymphula*
*Pyrrhosoma nymphula* (Sulzer, 1776)	BOLD:AAD5734	21	0.86	13.89	*L. albifrons*
Cordulegastridae					
*Cordulegaster boltonii* (Donovan, 1807)	BOLD:AAJ5773	4	0.62	8.61	*C. heros*
*Cordulegaster heros* Theischinger, 1979	BOLD:ACQ4796	12	0.87	8.61	*C. boltonii*
*Thecagaster bidentata* (Selys, 1843)	BOLD:AAJ5749	10	0.57	9.62	*C. boltonii*
Corduliidae					
*Cordulia aenea* (Linnaeus, 1758)	BOLD:AAJ5771	19	0.70	10.0	*S. alpestris*
*Epitheca bimaculata* (Charpentier, 1825)	BOLD:ACG0805	5	0.44	12.33	*A. alpestris*
*Somatochlora alpestris* (Selys, 1840)	BOLD:ACP5227	5	0.72	4.25	*S. arctica*
*Somatochlora arctica* (Zetterstedt, 1840)	BOLD:ACP7013	7	1.26	4.25	*S. alpestris*
*Somatochlora flavomaculata* (Vander Linden, 1825)	BOLD:AEC6167	15	1.19	7.83	*S. alpestris*
*Somatochlora meridionalis* Nielsen, 1935	BOLD:ABW6681	4	0.68	0.00	*S. metallica*
*Somatochlora metallica* (Vander Linden, 1825)	BOLD:ABW6681	22	1.34	0.00	*S. meridionalis*
Gomphidae					
*Gomphus pulchellus* Selys, 1840	BOLD:ADC4839	4	0.31	14.61	*O. cecilia*
*Gomphus vulgatissimus* (Linnaeus, 1758)	BOLD:AAN0925	8	0.84	15.40	*G. pulchellus*
*Onychogomphus forcipatus* (Linnaeus, 1758)	BOLD:AEM8698	9	2.84	10.96	*O. cecilia*
*Ophiogomphus cecilia* (Fourcroy, 1785)	BOLD:ACP4340	8	0.42	10.96	*O. forcipatus*
*Stylurus flavipes* (Charpentier, 1825)	BOLD:ADC2840	3	0.62	15.60	*O. forcipatus*
Lestidae					
*Chalcolestes parvidens* (Vander Linden 1825)	BOLD:AAI7225 BOLD:ADR7794	4	9.59	0.00	*C. viridis*
*Chalcolestes viridis* (Vander Linden 1825)	BOLD:AAI7225 BOLD:ADR7794	22	10.21	0.00	*C. parvidens*
*Chalcolestes parvidens* x *viridis*	BOLD:ADR7794	1	0.00	0.74	*C. viridis*
*Lestes barbarus* (Fabricius, 1798)	BOLD:ADC3442	5	0.28	11.07	*L. sponsa*
*Lestes macrostigma* (Eversmann, 1836)	BOLD:ADC3318	4	0.46	12.83	*L. sponsa*
*Lestes sponsa* (Hansemann, 1823)	BOLD:ACP4984	27	1.31	11.07	*L. barbarus*
*Lestes virens* (Charpentier, 1825)	BOLD:ACG0123	4	0.69	12.37	*L. sponsa*
*Sympecma fusca* (Vander Linden, 1820)	BOLD:AAK1032	9	0.74	7.73	*S. paedisca*
*Sympecma paedisca* (Brauer, 1877)	BOLD:ACG0335	1	0.00	7.73	*S. fusca*
Libellulidae					
*Crocothemis erythraea* (Brullé, 1832)	BOLD:AEX9418	13	1.45	13.23	*O. cancellatum*
*Leucorrhinia albifrons* (Burmeister, 1839)	BOLD:ADR0815	2	0.00	9.88	*L. caudalis*
*Leucorrhinia caudalis* (Charpentier, 1840)	BOLD:ADC4475	4	0.28	9.88	*L. albifrons*
*Leucorrhinia dubia* (Vander Linden, 1825)	BOLD:AAJ2437	10	0.56	2.30	*L. rubicunda*
*Leucorrhinia pectoralis* (Charpentier, 1825)	BOLD:ADC3719	4	0.00	4.55	*L. dubia*
*Leucorrhinia rubicunda* (Linnaeus, 1758)	BOLD:ADC1709	4	0.28	2.30	*L. dubia*
*Libellula depressa* Linnaeus, 1758	BOLD:AAJ2758	13	0.56	12.06	*L. fulva*
*Libellula fulva* Müller, 1764	BOLD:ACP3530	11	0.71	12.06	*L. depressa*
*Libellula quadrimaculata* Linnaeus, 1758	BOLD:AEY0197	18	0.59	12.42	*O. coerulescens*
*Orthetrum albistylum* (Selys, 1848)	BOLD:AED4117	13	0.28	5.33	*O. cancellatum*
*Orthetrum brunneum* (Fonscolombe, 1837)	BOLD:AAK5997	12	0.74	9.95	*O. cancellatum*
*Orthetrum cancellatum* (Linnaeus, 1758)	BOLD:AAK5996	18	2.37	5.33	*O. albistylum*
*Orthetrum coerulescens* (Fabricius, 1798)	BOLD:AAI2353	8	2.11	10.0	*O. brunneum*
*Sympetrum danae* (Sulzer, 1776)	BOLD:AAA3766	12	1.12	9.05	*S. depressiusculum*
*Sympetrum depressiusculum* (Selys, 1841)	BOLD:ACQ1493	4	0.31	9.05	*S. danae*
*Sympetrum fonscolombii* (Selys, 1840)	BOLD:AAI0218	5	0.85	14.34	*L. quadrimaculata*
*Sympetrum meridionale* (Selys, 1841)	BOLD:AAD8296	6	3.17	9.31	*S. sanguineum*
*Sympetrum pedemontanum* (Allioni, 1766)	BOLD:AAK1022	11	0.80	10.24	*S. vulgatum*
*Sympetrum sanguineum* (Müller, 1764)	BOLD:AAB2237	25	1.44	9.31	*S. meridionale*
*Sympetrum striolatum* (Charpentier, 1840)	BOLD:AAB2236	20	1.04	8.78	*S. vulgatum*
*Sympetrum vulgatum* (Linnaeus, 1758)	BOLD:AAE2658	25	1.0	8.78	*S. striolatum*
Platycnemididae					
*Platycnemis pennipes* (Pallas, 1771)	BOLD:ACG0515	30	0.44	16.91	*A. juncea*

## Data Availability

The data (COI and 16S sequences as well as the metadata) presented in this study are openly available in BOLD (DS-BAAZ) at https://dx.doi.org/10.5883/DS-BAAZ.

## Supplementary Materials

The following supporting information can be downloaded at https://www.mdpi.com/article/10.3390/insects16101056/s1; Table S1: number and origin of samples and sequences; Figure S1: Visualization of barcode gap; Figure S2: NJ tree of Austrian Anisoptera based on sequences of the whole *COI*; Figure S3: NJ tree of Austrian Zygoptera based on sequences of the whole *COI*; Table S2: intra- and interspecific distances based on *16S*.
