# Supplementary material for: A DNA Barcode Inventory of Austrian Dragonfly and Damselfly (Insecta: Odonata) Species
Source: Insects. 2025 Oct 16;16(10):1056. doi: 10.3390/insects16101056 (PMC12565296; doi:10.3390/insects16101056)
Supplement: Supplementary file 1 [file insects-16-01056-s001.zip › insects-3892928-supplementary/Figure S1.pdf]

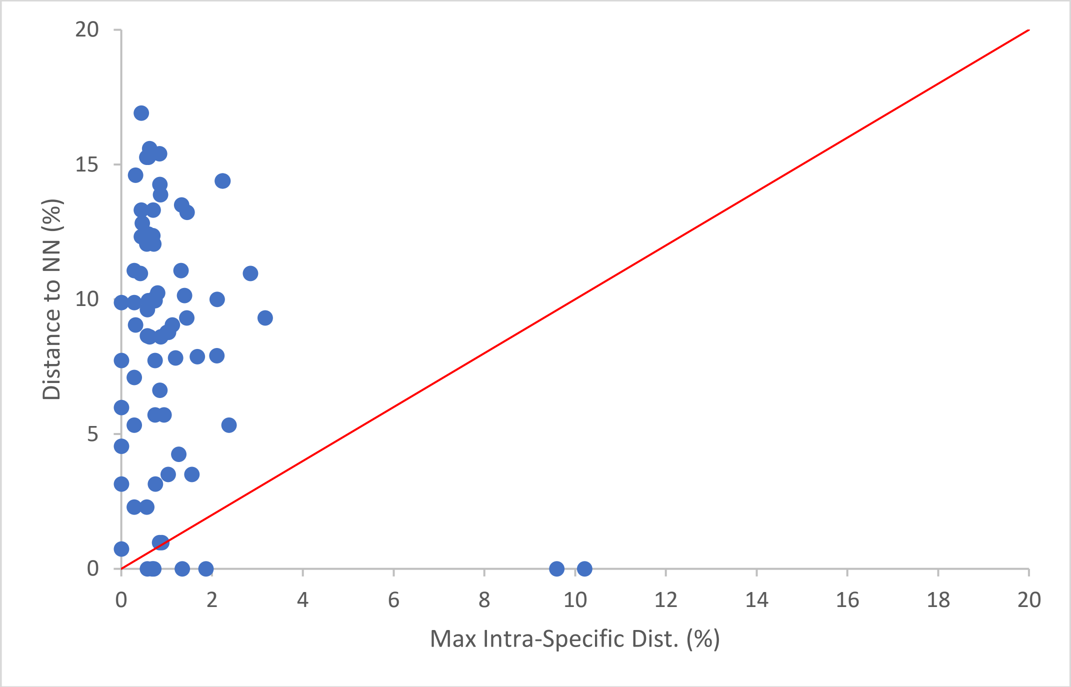

Figure S1. Visualization of the barcode gap present in all species except *Chalcolestes parvidens, C. viridis, Coenagrion ornatum, C. puella, C. pulchellum, Somatochlora meridionalis* and *S. metallica*. Retrieved from BOLD using the Barcode Gap Analysis tool (K2P distance model, pairwise deletion, BOLD aligner).
