# Supplementary material for: A DNA Barcode Inventory of Austrian Dragonfly and Damselfly (Insecta: Odonata) Species
Source: Insects. 2025 Oct 16;16(10):1056. doi: 10.3390/insects16101056 (PMC12565296; doi:10.3390/insects16101056)
Supplement: Supplementary file 1 [file insects-16-01056-s001.zip › insects-3892928-supplementary/Table S1.pdf]

Table S1: Info on number and origin of samples and sequences.

		No. of samples	No. of COI sequences	No. of 16S sequences
Dataset in total		892	839	867
Collected/generated in this study		839	786	867 (including 20 from [3] and 24 from [45])
Additional data	[45]	24	24	-
	[3]	29	29	-

for references see main text
