# Supplementary material for: A DNA Barcode Inventory of Austrian Dragonfly and Damselfly (Insecta: Odonata) Species
Source: Insects. 2025 Oct 16;16(10):1056. doi: 10.3390/insects16101056 (PMC12565296; doi:10.3390/insects16101056)
Supplement: Supplementary file 1 [file insects-16-01056-s001.zip › insects-3892928-supplementary/Table S2.pdf]

Table S2. The following table lists the number of samples per species (N) sequenced for *16S*, as well as mean inter- (distance to nearest neighbor, DNN_{Mean}) and intraspecific K2P distances (I_{Mean}) based on *16S* sequences between and within species, respectively.

**Species**	**N**	**I_{Mean} (%)**	**DNN_{Mean} (%)**
Aeshnidae			
Aeshna affinis	5	0	1.31
Aeshna caerulea	3	0	1.14
Aeshna cyanea	22	0.06	0.96
Aeshna grandis	8	0.3	0.49
Aeshna juncea	17	0	0.65
Aeshna mixta	14	0.27	0.37
Aeshna subarctica	4	0	0.37
Aeshna viridis	3	0	0.49
Anax ephippiger	2	0	0.21
Anax imperator	15	0.04	0.21
Anax parthenope	7	0.37	0.67
Brachytron pratense	8	0	0.98
Isoaeshna isoceles	11	0.33	1.64
Calopterygidae			
Calopteryx splendens	13	0.3	2.6
Calopteryx virgo	21	0.03	2.6
Coenagrionidae			
Coenagrion hastulatum	14	0	3.16
Coenagrion mercuriale	1	n/c	1.41
Coenagrion ornatum	6	0	0
Coenagrion puella	40	0.44	0.22
Coenagrion pulchellum	25	0	0
Coenagrion scitulum	6	0.19	3.44
Enallagma cyathigerum	28	0.25	6.91
Erythromma lindenii	4	0.14	5.12
Erythromma najas	13	0.16	3.79
Erythromma viridulum	18	0.09	3.79
Ischnura elegans	22	0.03	5.25
Ischnura pumilio	7	0	5.25
Nehalennia speciosa	8	0.22	5.9
Pyrrhosoma nymphula	21	0.08	5.9
Cordulegastridae			
Cordulegaster boltonii	3	0	1.31
Cordulegaster heros	12	0	1.31
Thecagaster bidentata	9	0.76	2.71
Corduliidae			
Cordulia aenea	18	0.04	3.8
Epitheca bimaculata	3	0	5.04
Somatochlora alpestris	5	0	3.06
Somatochlora arctica	7	0	3.06
Somatochlora flavomaculata	15	0.26	2.32
Somatochlora meridionalis	5	0.13	0.32
Somatochlora metallica	23	0.35	0.32
Gomphidae			
Gomphus pulchellus	4	0	3.37
Gomphus vulgatissimus	6	0.28	3.37
Onychogopmhus forcipatus	8	0.67	4.2
Ophiogomphus cecilia	5	0.13	4.2
Stylurus flavipes	2	0	4.33

Lestidae			
Chalcolestes parvidens	4	2.9	1.54
Chalcolestes viridis	24	0.27	1.54
Chalcolestes parvidens x viridis	1	n/c	1.67
Lestes barbarus	6	0	4.04
Lestes macrostigma	4	0	4.92
Lestes sponsa	40	0.14	3.41
Lestes virens	9	0.06	3.41
Sympecma fusca	12	0	3.45
Sympecma paedisca	1	n/c	3.45
Libellulidae			
Crocothemis erythraea	12	0.15	6.49
Leucorrhinia albifrons	2	0	2.72
Leucorrhinia caudalis	4	0.16	2.72
Leucorrhinia dubia	10	0	0.65
Leucorrhinia pectoralis	4	0	0.32
Leucorrhinia rubicunda	4	0	0.32
Libellula depressa	13	0	3.69
Libellula fulva	10	0.06	3.69
Libellula quadrimaculata	19	0	3.99
Orthetrum albistylum	13	0.12	0.56
Orhtetrum brunneum	12	0	4.67
Orthetrum cancellatum	17	0.3	0.56
Orthetrum coerulescens	8	0.67	3.4
Sympetrum danae	12	0.05	3.62
Sympetrum depressiusculum	5	0	3.62
Sympetrum fonscolombii	8	0.22	3.69
Sympetrum meridionale	6	0	3.31
Sympetrum pedemontanum	7	0.18	4.47
Sympetrum sanguineum	25	1.04	3.78
Sympetrum striolatum	21	0	2.34
Sympetrum vulgatum	26	0.07	2.34
Platycnemididae			
Platycnemis pennipes	32	0.34	10.93
