# Supplementary figures and images for: A DNA Barcode Inventory of Austrian Dragonfly and Damselfly (Insecta: Odonata) Species
Source: Insects. 2025 Oct 16;16(10):1056. doi: 10.3390/insects16101056 (PMC12565296; doi:10.3390/insects16101056)

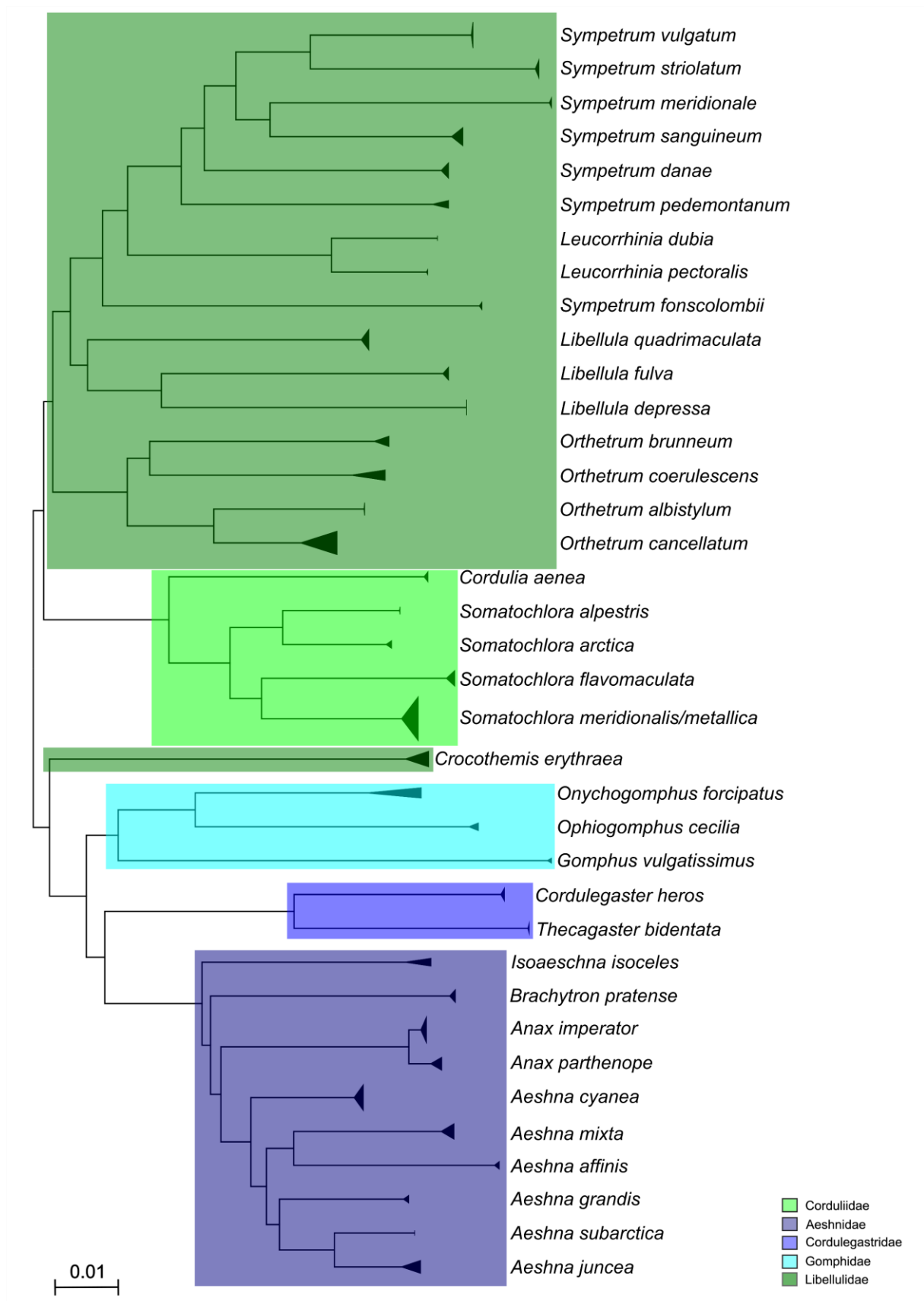

Figure S2. NJ tree of Austrian Anisoptera based on sequences of the whole COI gene.

Supplement: Supplementary file 1 [file insects-16-01056-s001.zip › insects-3892928-supplementary/Figure S2.pdf]

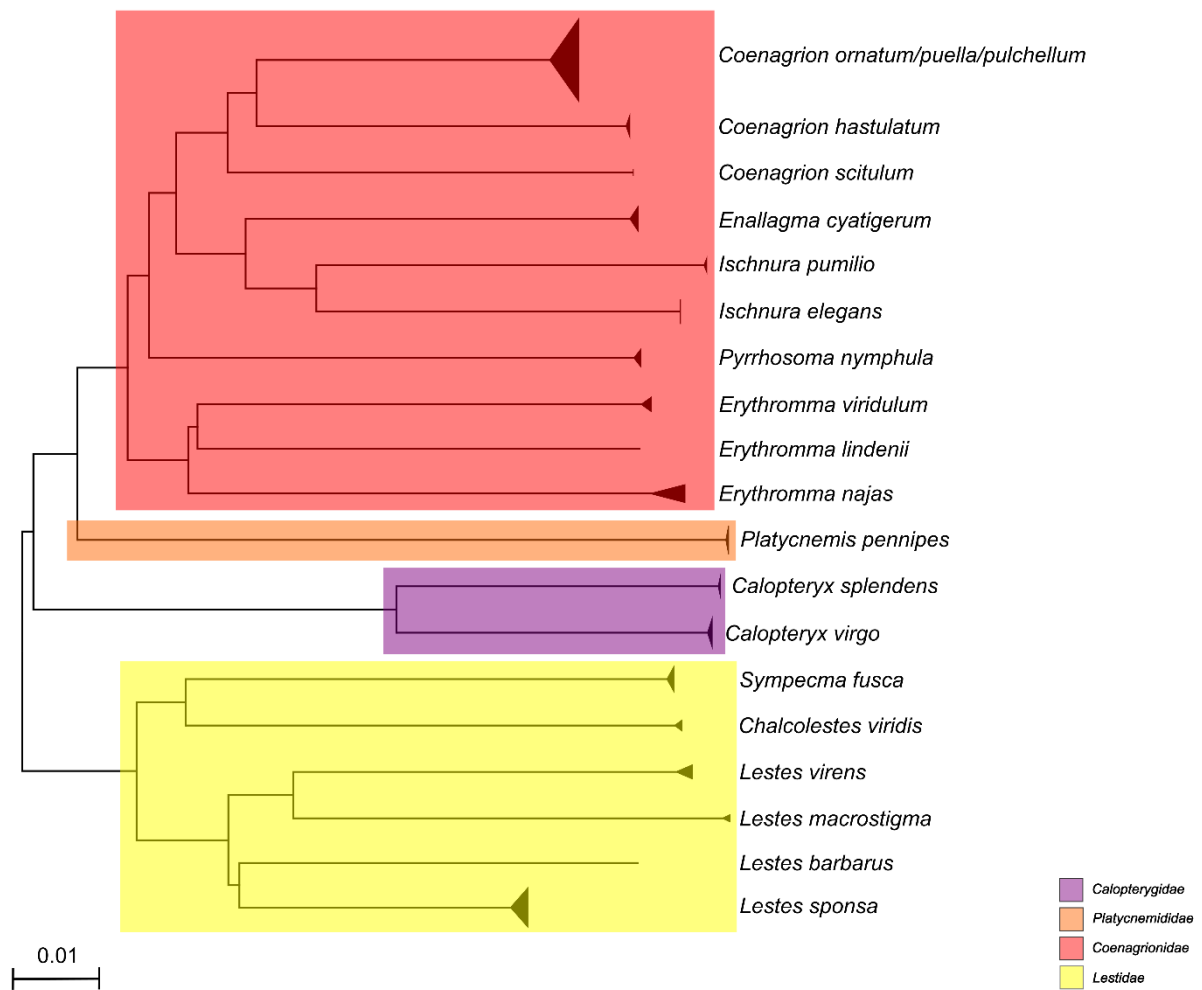

**Figure S3.** NJ tree of Austrian Zygoptera based on sequences of the whole *COI* gene.

Supplement: Supplementary file 1 [file insects-16-01056-s001.zip › insects-3892928-supplementary/Figure S3.pdf]
